# Varied Responses to a High m.3243A>G Mutation Load and Respiratory Chain Dysfunction in Patient-Derived Cardiomyocytes

**DOI:** 10.3390/cells11162593

**Published:** 2022-08-19

**Authors:** Sanna Ryytty, Shalem R. Modi, Nikolay Naumenko, Anastasia Shakirzyanova, Muhammad Obaidur Rahman, Miia Vaara, Anu Suomalainen, Pasi Tavi, Riikka H. Hämäläinen

**Affiliations:** 1 A. I. Virtanen Institute for Molecular Sciences, University of Eastern Finland, 70211 Kuopio, Finland; 2 Stem Cell and Metabolism Research Program, Research Programs Unit, University of Helsinki, 00290 Helsinki, Finland; 3 HUSLab, Helsinki University Hospital, 00290 Helsinki, Finland

**Keywords:** mitochondria, cardiomyopathy, mtDNA, iPS-cell, cardiomyocyte, respiratory chain dysfunction

## Abstract

The m.3243A>G mutation in mitochondrial *tRNA-Leu(UUR)* is one of the most common pathogenic mitochondrial DNA mutations in humans. The clinical manifestations are highly heterogenous and the causes for the drastic clinical variability are unknown. Approximately one third of patients suffer from cardiac disease, which often increases mortality. Why only some patients develop cardiomyopathy is unknown. Here, we studied the molecular effects of a high m.3243A>G mutation load on cardiomyocyte functionality, using cells derived from induced pluripotent stem cells (iPSC-CM) of two different m.3243A>G patients, only one of them suffering from severe cardiomyopathy. While high mutation load impaired mitochondrial respiration in both patients’ iPSC-CMs, the downstream consequences varied. mtDNA mutant cells from a patient with no clinical heart disease showed increased glucose metabolism and retained cellular ATP levels, whereas cells from the cardiac disease patient showed reduced ATP levels. In this patient, the mutations also affected intracellular calcium signaling, while this was not true in the other patient’s cells. Our results reflect the clinical variability in mitochondrial disease patients and show that iPSC-CMs retain tissue specific features seen in patients.

## 1. Introduction

Mitochondrial dysfunction is a common cause of human disease with an estimated minimum prevalence of 1 in 5000 adults [1]. The patients present vast clinical and genetic heterogeneity, with a common nominator of a primary defect in the mitochondrial respiratory chain (RC) function. This can be the result of an inherited mutation in the nuclear genome or in the mitochondrial genome (mtDNA), as mitochondria possess their own multi-copy genome encoding 13 subunits of the respiratory chain proteins and the tRNAs (transfer ribonucleic acids) and rRNAs (ribosomal ribonucleic acids) required to translate these proteins. Clinical manifestations in patients are most often seen in tissues with high energy demand, and neurological defects, which include neuropathy, ataxia, cognitive problems, stroke-like episodes and/or epilepsy, are commonly present. Many patients also manifest muscle disease, with myopathy, muscle weakness and exercise intolerance as common symptoms [2]. Cardiomyopathy, which significantly increases the mortality rate, is a relatively common manifestation in mitochondrial disease, which is not surprising, considering the high energetic demand and contractile and oxidative activity of cardiomyocytes. Why some mitochondrial diseases affect the heart and others do not, is an open question in the field. Typical cardiac manifestations in mitochondrial disease patients include hypertrophic and dilated cardiomyopathy, arrhythmias, left ventricular myocardial noncompaction, and heart failure [3].

Heteroplasmic m.3243A>G mutation in the *tRNA-Leu(UUR)* gene (*MTTL1*) is one of the most common human disease causing mtDNA mutations [4,5], with a prevalence of 1/6000 in the Finnish population [6] and an estimated carrier frequency of 1/400 in the Caucasian population [7]. As typical for mitochondrial disease, patients with this mutation present various clinical phenotypes. Mitochondrial encephalopathy, lactic acidosis and stroke-like episodes (MELAS), maternally inherited diabetes and deafness (MIDD), myopathy, short stature, gastrointestinal symptoms, and cardiomyopathy are the major phenotypes seen in them [8,9]. Hypertrophic cardiomyopathy is present in 20–40% of patients carrying the m.3243A>G mutation [10].

Most human disease causing mtDNA mutations are heteroplasmic, meaning that both a healthy and a mutant form of mtDNA is present in patients’ cells. The heteroplasmic nature of the m.3243A>G mutation, and the variation in the amount of mutant mtDNA present in patients’ cells [11] may partially explain the remarkable clinical variation among the patients. For example, one study showed that the mtDNA mutation load correlates inversely with left ventricular glucose uptake in the hearts of m.3243A>G patients [12]. However, no clear correlation with the myocardial mutation load and severity of the clinical cardiomyopathy or mitochondrial defects in patients’ histological samples were found in recent studies [13,14], suggesting that the amount of mutant mtDNA does not alone dictate the clinical outcome of the m.3243A>G cardiomyopathy. Thus, why only a proportion of the mutation carriers develop clinical heart disease is not understood. Similarly, while the cardiac involvement in m.3243A>G carriers is evident and seen in systemic studies [15], the molecular mechanisms leading to cardiac disease remains unknown. Due to unknown disease mechanisms, current treatment options for mitochondrial cardiomyopathies are limited and only rely on relieving symptoms.

To investigate the effect of the m.3243A>G mutation load on defining the functional cardiac cell phenotype, we studied cardiomyocytes derived from induced pluripotent stem cells (iPSCs) of two m.3243A>G patients. Both patients manifested MIDD-like phenotypes accompanied by severe cardiomyopathy in one of them (P2, CMP patient), while no cardiac symptoms were present in the other patient (P1, MIDD patient) [16]. Interestingly, while a high mtDNA mutation load caused a similar defect in the respiratory chain function in both patients’ cells, the metabolic consequences are different between these patients. Functional consequences are present only in the cells of the patient with clinical heart disease, indicating that a high mutation load alone does not dictate the functional outcome in patients’ cardiac cells.

## 2. Materials and Methods

### 2.1. Patient Cells and Induced Pluripotent Stem Cell Culture

iPSCs, included in the study, were earlier generated from fibroblasts from two patients carrying the m.3243A>G mutation [16]. The mutation amounts in the parental patient 1 and 2 fibroblasts were 22% and 35%, respectively. The appropriate ethical permits were obtained from the Coordinating Ethics Committee of the Helsinki and Uusimaa Hospital District and the material was utilized by their informed consent. P1, a 39-year-old man, presented clinically with MIDD and ataxia, but the ECG (electrocardiography) was normal, whereas a 55-year-old woman (P2), suffered from severe cardiomyopathy as well as MIDD-like symptoms. Family history and the clinical phenotype supported mitochondrial origin for the CMP, thus no other CMP genes were screened for. Two independent iPSC lines were analyzed for the high mutation load group of P2 (mutation loads 60% and 80%), whereas three independent cell lines were included in all other groups. P1 high mutation (75–85%), P1 control (0.5–1.5%), P2 control (0.5–3%). The iPSCs were maintained in Essential 8 medium on growth factor reduced Matrigel (Corning, New York, NY, USA). The media was supplemented with 0.05 mg/mL uridine at all stages of the *in vitro* culture. The cells were passaged every 3–5 days using EDTA (ethylenediaminetetraacetic acid). iPSC lines were used for differentiation between passages 20 and 60. 

### 2.2. Differentiation and Characterization of Cardiomyocytes

Cardiomyocytes (CMs) were differentiated from iPSCs utilizing a previously published protocol based on temporal modulation of Wnt/β-catenin signaling [17]. Briefly, after single cell seeding, the cells were first treated with GSK3 (glycogen synthase kinase-3) inhibitor (9 µM CHIR 99021, Tocris, Bristol, UK) for 1 day to induce mesoderm differentiation. Two days after withdrawal of the GSK3 inhibitor, Wnt signaling inhibitor (5 µM IWP2, Tocris, Bristol, UK) was added to the culture media for two days to induce cardiac mesoderm. After 4 weeks, contracting CMs were collected manually (individual areas were hand-picked) and maturated for 5 days in high glucose DMEM (Dulbecco’s Modified Eagle Medium) with 10% FBS (Fetal Bovine Serum, Gibco, Waltham, MA, USA). After maturation, contracting cardiomyocytes were collected manually.

### 2.3. Flow Cytometry

For flow cytometry, cells were dissociated to single cells with collagenase-pancreatin solution (Collagenase Type II (Worthington) 630 U/mL, Pancreatin (Sigma-Aldrich, Burlington, MA, USA) 0.2 mg/mL). A total of 100,000 cells were collected per sample. To assess the presence of cardiac cells in samples, the cells were fixed with 2% paraformaldehyde in PBS (Phosphate Buffered Saline) for 10 min on ice, permeabilized with 0.5% saponin in PBS for 15 min, washed with 0.1% saponin in PBS, and stained using 1:50 anti-cardiac troponin T-FITC (Fluorescein 5-isothiocyanite) antibody (Miltenyi Biotec, Bergisch Gladbach, Germany, 130-106-687) in 5% FBS, 0.1% saponin in PBS for 30 min. Cells were further washed and suspended to 1% FBS in PBS.

For analyzing mitochondrial membrane potential, cells were stained with 20 nM Tetramethylrhodamine Methyl Ester Perchlorate (TMRM, Thermo Scientific, Waltham, MA, USA) and 200 nM MitoTracker Green (Invitrogen, Carlsbad, CA, USA) for 10 min at +37 °C. Cells were then washed twice with medium, the second wash included 1:1500 DAPI (4′6′-diamidino-2-phenylindole), then they were suspended in 200 µL 1% FBS in DMEM. 

For the analysis of glucose uptake, cells were incubated with 100 µM 2-(N-(7-Nitrobenz-2-oxa-1,3-diazol-4-yl) Amino)-2-Deoxyglucose (2-NBDG; Thermo Fischer, Waltham, MA, USA) in glucose-free RPMI (Roswell Park Memorial Institute) medium at 37 °C for 30 min. Cells were then washed twice with glucose-free RPMI, the second wash included 1:1500 DAPI, and suspended in glucose-free RPMI. All cytometric analysis was done with Cytoflex S Analyzer (Beckman Coulter, Brea, CA, USA), and the data was analyzed using CytExpert (Version 2.3, Beckman Coulter, Brea, CA, USA). 

### 2.4. Seahorse Analysis for Cellular Bioenergetics

Oxygen consumption rate (OCR) and extracellular acidification rate (ECAR) were assessed with XF96 Extracellular Flux Analyzer (Seahorse Bioscience, North Billerica, MA, USA). Differentiated cardiomyocytes were seeded in maturation medium at a density of 5000 cells per well, 5 days before the run. On the day of the run, the cells were washed with assay medium and incubated for 1h in assay medium (including glucose, pyruvate, and glutamine) at +37 °C without CO2. OCR and ECAR levels were assessed from the change in oxygen and H^+^ concentration over time. After measuring baseline OCR and ECAR, sequential automatic injections of a final concentration of 1 μM oligomycin (an ATP (adenosine triphosphate) synthase inhibitor, Sigma-Aldrich, Burlington, MA, USA), 1 μM carbonyl cyanide p-[trifluoromethoxy]-phenylhydrazone (FCCP, a mitochondrial uncoupler, Sigma-Aldrich, Burlington, MA, USA), and 1 μM antimycin A (complex III inhibitor, Sigma-Aldrich, Burlington, MA, USA) together with 1 μM rotenone (complex I inhibitor, Sigma-Aldrich). The data was normalized to total protein content, analyzed with Pierce BCA (bicinchoninic acid) Protein Assay (Thermo Scientific, Waltham, MA, USA). The results were analyzed with Wave program (Agilent Technology, Santa Clara, CA, USA).

### 2.5. Immunocytochemistry

For immunocytochemistry, the cells were fixed with 4% paraformaldehyde for 20 min at room temperature (RT). For permeabilizing and blocking, cells were then incubated in 0.2% TritonX-100 and 10% normal swine serum (NSS) (DAKO, Santa Clara, CA, USA, X0901) in PBS for 1 h at RT. Primary antibody was diluted in 0.2% TritonX-100 and 1% NSS in PBS and then cells were incubated overnight at 4 °C. Secondary antibody was diluted in 0.2% TritonX-100 and 1% NSS in PBS and then cells were incubated 1–2 h at RT. Cells were washed with PBS. Nuclear staining (DAPI) was added in a last wash (0.7 μg/mL for 5 min). Cells were imaged with Zeiss AXIO Observer Z1 (Jena Germany), with 40× objective, using excitation of 488 nm for green, 594 nm for red, and 405 nm for blue (DAPI). Used antibodies and their dilutions are presented in Table A1.

### 2.6. Calcium Imaging 

Calcium imaging was performed as previously described [18]. Briefly, cardiomyocytes were loaded with Fluo-4-acetoxymethyl (AM)-ester (5 μM; 0.02% pluronic acid, Invitrogen, Carlsbad, CA, USA) in DMEM for 30 min. The cells were then placed in a recording chamber (Cell MicroControls, flow rate approx. 1–2 mL/min, chamber volume 0.4 mL) and continuously perfused with DMEM bubbled with carbogen gas. Measurements were carried out after an incubation period of 20 min to allow de-esterification of the dye. All experiments were carried out at 37 °C sustained by a temperature controller (TC2BIP, Cell MicroControls). Measurements were performed with a confocal inverted microscope (FluoView 1000; Olympus, Tokyo, Japan). The cells were excited at 488 nm and the emitted light was collected at 500–600 nm through 60× objective lens and in line-scan mode. To excite the cells, myocytes were stimulated with 1-ms voltage pulses 50% over the excitation threshold through two platinum wires located on both sides of the chamber. Caffeine (10 mM, Sigma-Aldrich, Burlington, MA, USA) was applied directly to the studied area with a local perfusion manifold (Cell MicroControls). The images were analyzed with FluoView software and ImageJ (imagej.nih.gov/ij/) software. Fluo-4 fluorescence intensity is shown as F/F-ratio, where F is the background subtracted fluorescence intensity and F0 is background subtracted minimum fluorescence value measured from each cell at rest. P1 and P2 were measured and analyzed independently. 

### 2.7. ROS Analysis

Cells were loaded in 5 μM MitoSOX Red (M36008, Invitrogen, Carlsbad, CA, USA) in DMEM media for 20 min, placed in a recording chamber, and measured as described above for calcium imaging. Frame scan confocal images were obtained every 30 s at emitted light band 550–650 nm through 20× objective lens. After 30 min, FCCP (1 µM, Carbonyl cyanide 4-(trifluoromethoxy)phenylhydrazone) was applied directly to the studied area. Changes in fluorescence intensity were calculated in each responding cell off-line and are expressed as arbitrary fluorescence units (AFU). P1 and P2 were measured and analyzed separately. 

### 2.8. DNA Extraction 

Total DNA was extracted with DNA lysis buffer (100 mM Tris-HCL, 5 mM EDTA, 0.2% SDS (sodium dodecyl sulfate), 200 mM NaCl) and Proteinase K (100 µg/mL, Thermo Scientific, Waltham, MA, USA) at 55 °C overnight. The next day, an equal volume of isopropanol was added, and samples were shaken until DNA precipitate became visible. The samples were centrifuged at 10,000 g for 5 min to pellet the DNA, which was further washed twice with 70% ethanol. After air drying at RT for 15 min, the DNA was resuspended in 50 μL of nuclease-free water and incubated at 55 °C for 30 min to dissolve. The DNA concentration was measured with a NanoDrop ND-1000 Spectrophotometer. 

### 2.9. Quantitative PCR 

The mitochondrial copy number was determined from the total DNA by measuring relative quantity of the mitochondrial *CYTB* (cytochrome b) gene against the nuclear *APP* (amyloid precursor protein) gene in a SYBR green assay (Table A2). A qPCR was performed with 25 ng of DNA using the Maxima SYBR green Master Mix (Thermo Scientific, Waltham, MA, USA) with a Step One Plus PCR machine (Applied Biosystem, Waltham, MA, USA). 

Gene expression was determined from RNA extracted with TRIzol reagent (Invitrogen). A total of 500 ng of RNA was used for cDNA synthesis; cDNA was diluted 1:20 for qPCR, which was performed using the Maxima SYBR green Master Mix (Thermo Scientific, Waltham, MA, USA). Delta-Delta CT method [19] was used for analyzing the results. The primer sequences are presented in Table A2. All qPCR reactions were done in triplicates.

### 2.10. Determination of the m.3243A>G Mutation Load

The m.3243A>G mutation load was determined from total DNA by measuring the relative quantity of the m.3243A allele against the m.3243G allele in qRT-PCR in a Taqman assay (primer and probe sequences presented in Table A3). The PCR was run with Maxima probe/ROX (carboxy-x-rhodamine) qPCR master mix (Thermo Scientific, Waltham, MA, USA) in Step One Plus machine (Applied Biosystem, Waltham, MA, USA). For analysis, a calibration curve was created using standards with plasmids containing amplified mtDNA fragments of either wild-type or mutant allele or the two of them. All reactions were run in triplicates.

### 2.11. Western Blotting

Total proteins were isolated with RIPA (radioimmunoprecipitation assay) lysis buffer and protease inhibitors after 15 min incubation on ice. A total of 10–15 µg of total protein from each lysate was loaded onto a 10–12% Tris Gel. The gel was transferred onto a PVDF (polyvinylidene fluoride) membrane, which was then blocked by 5% milk for 1 h at RT. The membrane was washed once and incubated in primary antibody diluted in 5% BSA in PBS overnight at 4 °C. The next day, the membrane was washed three times with PBS-T, incubated with secondary antibody for 1 h at RT, washed again three times with PBS-T, and processed with ECL (enhanced chemiluminescence) substrate (Pierce). Membranes were imaged with the ChemiDoc MP imaging system (Bio-Rad, Hercules, CA, USA) and analyzed with Image Lab (Bio-Rad, Hercules, CA, USA, version 5.1). Used antibodies and their dilutions are presented in Table A1.

### 2.12. ATP Concentration

Intracellular ATP levels of iPSC-CMs were quantified using an ATPlite assay kit according to the manufacturer’s instructions (PerkinElmer, Waltham, MA, USA). In total, 50,000 cells per sample were collected on 96-well microplates with cell lysis solution. After shaking for 5 min at RT, an equal amount of substrate was added and mixed for another 5 min at RT. The luminescent intensities were monitored after 10 min with Victor2 1420 multilabel counter (Wallac, Turku, Finland). 

### 2.13. Lactate Concentration

Intracellular lactate levels of iPSC-CMs were quantified with Lactate Assay kit (Sigma-Aldrich, Burlington, MA, USA). A total of 100,000 cells per sample were collected on 96-well microplates with lysis solution. After shaking, an equal amount of substrate was added and mixed for 15 min at RT. The luminescent intensities were monitored with Victor2 1420 multilabel counter (Wallac, Turku, Finland). 

### 2.14. Statistical Analysis

The data were analyzed using GraphPad Prism. The data are presented as mean ± SD. The statistical significance was determined with ANOVA (one-way) and Student’s t-test (unpaired and two-tailed) and *p* < 0.05 was considered as significant.

## 3. Results

### 3.1. Cells with High mtDNA Mutation Load Show Normal Cardiac Differentiation Potential

**Figure 1:**
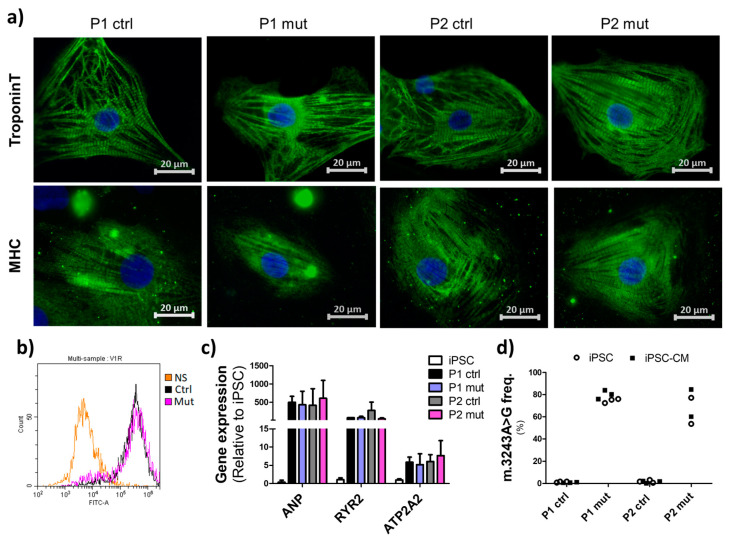
Characterization of iPSC-CMs. (**a**) Representative images of immunostaining for cardiac structural proteins cardiac troponin and Myosin Heavy Chain (MHC) (green) showing typical iPCS-CM morphology. Nuclear counter staining with DAPI (blue). Scalebars 20 µm. (**b**) Representative histograms from flow cytometry analysis for cardiac troponin T positive cells in iPSC-CM cultures with approximately 80% of the cells showing cardiac identity. NS = nonstained = no primary antibody. (**c**) Relative expression of cardiac specific genes in iPSC-CMs compared to iPSCs. Results are normalized against *GAPDH* (glyceraldehyde-3-phosphate dehydrogenase) expression and shown as mean +/ࢤ SD relative to one control line. Data from four independent experiments, *n* = 8. (**d**) Representative results from the quantitative PCR analysis of the m.3243A>G mutation load in the parental iPSCs and after cardiac differentiation (iPSC-CM) revealing stable mutation levels during differentiation.

Previously generated iPSCs from two patients were utilized in this study [16]. To focus on the effect of the m.3243A>G mutation load and exclude biological variation between individuals, we compared cells with a high mutation amount (60–85%, mut) to cells originating from the same patient with negligible mutation loads (<3%, ctrl). Multiple lines per group were included to account for clone dependent variation. To differentiate the iPSCs to cardiomyocytes (iPS-CMs) we adopted a previously published method utilizing modulation of Wnt/β-catenin signaling [17]. The cells started to contract spontaneously after 1–2 weeks of differentiation and after 4 weeks, the contracting cells were mechanically collected and transferred to maturation medium. Spontaneous contraction continued during the whole maturation period. Immunostaining for cardiac structural proteins, human cardiac troponin-T and myosin heavy chain (MHC), revealed typical iPSC-CM morphology and sarcomere structures in all cell lines (Figure 1a) and flow cytometry analysis verified that after the manual selection, 75–90% of the cells in the iPSC-CM cultures were positive for cardiac troponin T (Figure 1b). We did not detect any evidence of decreased cardiac identity in cultures with high mutation loads. After maturation, expression levels of cardio-specific genes: *ANP* (atrial natriuretic peptide), *ATP2A2* (ATPase Sarcoplasmic/Endoplasmic Reticulum Ca^2+^ Transporting 2), and *RYR2* (ryanodine receptor 2) were upregulated in differentiated cells when compared to iPSCs, with no apparent differences in the expression levels between the lines (Figure 1c). All iPSC-CMs showed contractile activity and responded to electrical stimulation. The m.3243A>G mutation load was stable during differentiation (Figure 1d). These findings show that all iPSC lines were able to differentiate into phenotypically and functionally typical iPSC-CMs, with no evidence of the mtDNA mutations inducing defects in differentiation or maturation to CMs. Further, the identity of the cells was comparable between all cultures. 

### 3.2. Mitochondrial Mutations Do Not Induce Drastic Effects on Mitochondrial Mass or Morphology in iPSC-CMs

**Figure 2:**
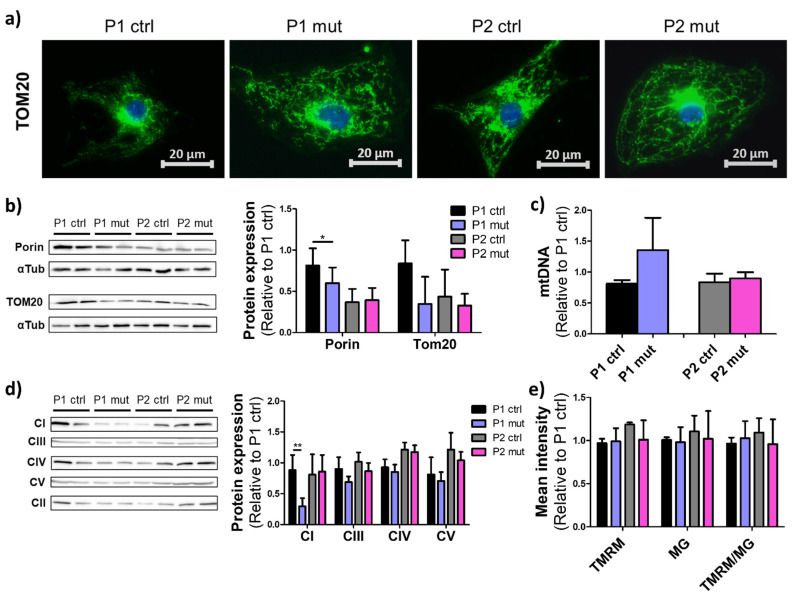
Mitochondrial mutations did not induce changes in mitochondrial mass or morphology. (**a**) Mitochondrial morphology presented with immunostaining of mitochondrial import receptor subunit TOM20 (green) in iPSC-CMs, representative images. Nuclear counter staining with DAPI (blue). Scalebars 20 µm. (**b**) Western blot analysis of mitochondrial membrane proteins Porin and TOM20. Normalization to α-Tubulin (αTub) protein levels, relative to one P1 ctrl line. Representative images and quantification of the data from 4 (Porin, *n* = 8) or 2 (TOM20, *n* = 4) independent blots. (**c**) mtDNA copy number analyzed by qPCR and normalized against nuclear DNA. Relative results to one P1 ctrl line, three independent experiments, *n* = 6. (**d**) Western blot analysis for mitochondrial respiratory chain proteins with CI, CIII, CIV, and CV levels normalized against CII levels. Analysis relative to one P1 ctrl line. Quantification from 3 independent blots, *n* = 6. Significant reduction of CI level is seen in mutant P1 cells. (**e**) Mean intensity of mitochondrial membrane potential marker TMRM (Tetramethylrhodamine), mitochondrial mass marker MG (MitoGreen) and their ratio in iPSC-CMs analyzed with flow cytometry. Three independent experiments, *n* = 6. * *p* < 0.05 ** *p* < 0.01. All results are shown as mean +/− SD.

Mitochondria are dynamic organelles with flexible morphology, and they undergo rapid transition according to the metabolic requirements of the cell [20]. To assess the effect of the m.3243A>G load on mitochondrial mass and morphology in the iPSC-CMs, we performed immunostaining for mitochondrial import receptor subunit TOM20 (Figure 2a). We did not observe any significant differences in mitochondrial morphology between the cells with different mutation amounts. However, western blot analysis suggested decreased levels of mitochondrial membrane protein porin in the MIDD patient (P1) lines with a high mutation load when compared to the isogenic control lines from the same patient, whereas no significant changes were seen between the lines of the CMP patient (P2) (Figure 2b, Supplementary Figure S1). The mtDNA copy number was unchanged in both patients’ lines (Figure 2c). 

To assess the levels of the OXPHOS components, western blot analysis was performed for the RC complexes (CI-CV). A decrease in the complex I (CI, NADH:ubiquinone oxidoreductase) level was seen upon a high mutation load only in the MIDD patient’s lines when compared to the same patient’s isogenic control lines. No difference was seen in the other RC complex proteins studied, CIII (ubiquinol:cytochrome c oxidoreductase), CIV (cytochrome c oxidase), or CV (ATP synthase) (Figure 2d, Supplementary Figure S1). All other RC complexes were normalized against CII (succinate dehydrogenase, SDH) levels. 

Mitochondrial membrane potential generated by the RC is crucial for ATP production and a common indicator of mitochondrial defects [21]. We did not detect any significant changes in mitochondrial membrane potential between iPSC-CM lines with a high or a low mutation load (Figure 2e). However, due to the limited sensitivity of the analysis methodology, minor changes cannot completely be ruled out. Further, despite the lower expression of mitochondrial membrane proteins in P1 mutant cells, no difference was observed between the lines when analyzing Mitotracker green staining by FACS (Figure 2e), suggesting that the mutations did not induce significant changes in mitochondrial mass.

These results show that mitochondrial mass or morphology are not significantly changed upon increasing mutation load. However, in the MIDD patient’s cells, a clear CI deficiency is seen upon a high mutation load. Similar change is not present in the cells of the CMP patient.

### 3.3. High m.3243A>G Mutation Load Impairs Oxidative Respiration in iPSC-CMs

**Figure 3:**
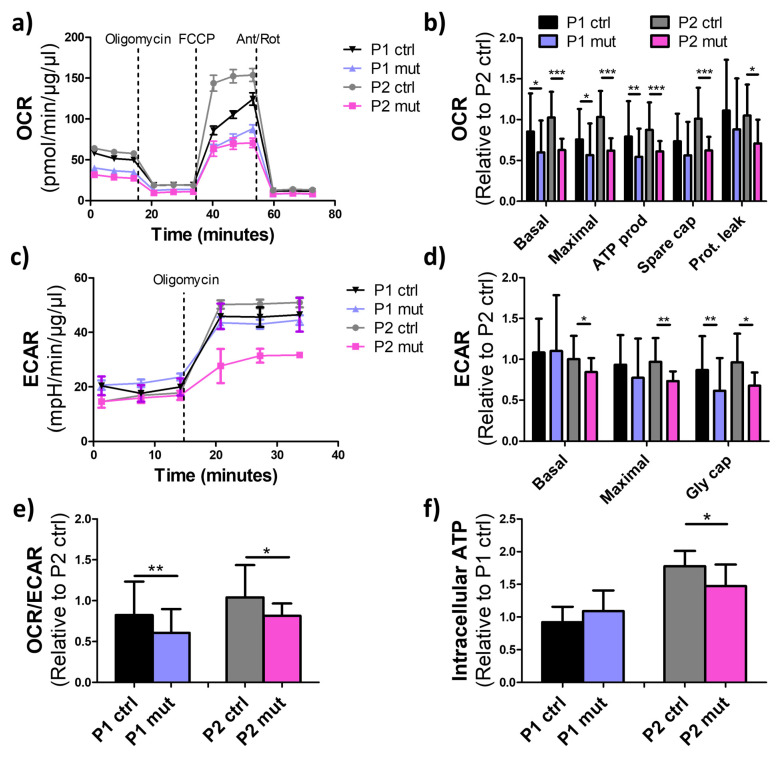
Mitochondrial respiration is decreased in patient cardiomyocytes upon a high mutation load. (**a**) Oxygen consumption rates (OCR) at basal level and after inhibition of the RC with oligomycin (CV inhibitor), FCCP (uncoupling agent), and Antimycin A (CIII inhibitor) and Rotenone (CI inhibitor) (Ant/Rot). Data is normalized to total protein content. Respiratory activity is reduced in mutant cells of both patients. (**b**) Analysis of the OCR data relative to one P1 ctrl line. Prot. Leak = Proton leak. (**c**) Extra Cellular Acidification Rates (ECAR) at the basal level and after inhibition of mitochondrial ATP synthesis with oligomycin, data normalized to total protein content. The ECAR is reduced in mutant cells of P2, indicating reduced glycolytic activity. (**d**) Analysis of the ECAR data relative to one P2 ctrl line. Gly cap = glycolytic capacity. (**e**) OCR/ECAR ratios representing relative oxidative activity calculated at basal level and relative to one P2 ctrl line. (**f**) Intracellular ATP levels in iPSC-CMs measured with ATPlite assay. Relative to one P1 ctrl line. Cellular ATP levels are reduced upon a high mutation amount in P2 cells. * *p* < 0.05 ** *p* < 0.01 *** *p* < 0.001. All results are shown as mean +/− SD and calculated from three independent experiments. Mitochondrial respiration analysis: P1 ctrl *n* = 36, P1 mut *n* = 38, P2 ctrl *n* = 74, P2 mut *n* = 14; ATPlite assay: P1 ctrl *n* = 20, P1 mut *n* = 13, P2 ctrl *n* = 15, P2 mut *n* = 28.

To assess the effect of the m.3243A>G mutation load on cellular respiration we analyzed the oxygen consumption rate (OCR) of the iPSC-CMs with SeahorseXF-96 Extracellular Flux Analyzer. In both patients’ cells, a high amount of the m.3243A>G mutation significantly decreased both basal and maximal respiration as well as oxidative ATP production (Figure 3a,b). Extra Cellular Acidification Rate (ECAR), which can be equated to represent the glycolytic rate of the cells, was also determined. The ECAR values suggested that high amount of the m.3243A>G mutation decreased both basal and maximal glycolysis in iPSC-CMs of the CMP patient (P2) but not in the MIDD patient (P1) (Figure 3c,d). However, upon inhibition of the mitochondrial ATP synthetase, control cells from both patients increased their glycolytic activity more than the mutant cells, suggesting that inhibition of RC function had more drastic effects in the control than in the mutant cells. In both patients, iPSC-CMs with high m.3243A>G mutation levels produced less energy by oxidative respiration than by glycolysis when compared to control iPSC-CMs (Figure 3e). In line with the reduced respiration, intracellular ATP levels, determined using an ATPlite assay, were reduced in the CMP patient’s (P2) iPSC-CMs with a high m.3243A>G mutation load when compared to control cells. However, in the MIDD patient (P1), no significant changes to cellular ATP levels were induced by the mutations (Figure 3f).

Together, these results show reduced oxidative metabolism in mutant cells of both patients. However, in the CMP patient’s mutant cells, glycolysis was also downregulated, suggesting overall suppressed metabolism leading to reduced ATP levels in mutant cells of this patient.

### 3.4. Compensatory Glucose Metabolism Is Upregulated in the MIDD Patient’s Cells upon High Mutation Load

**Figure 4:**
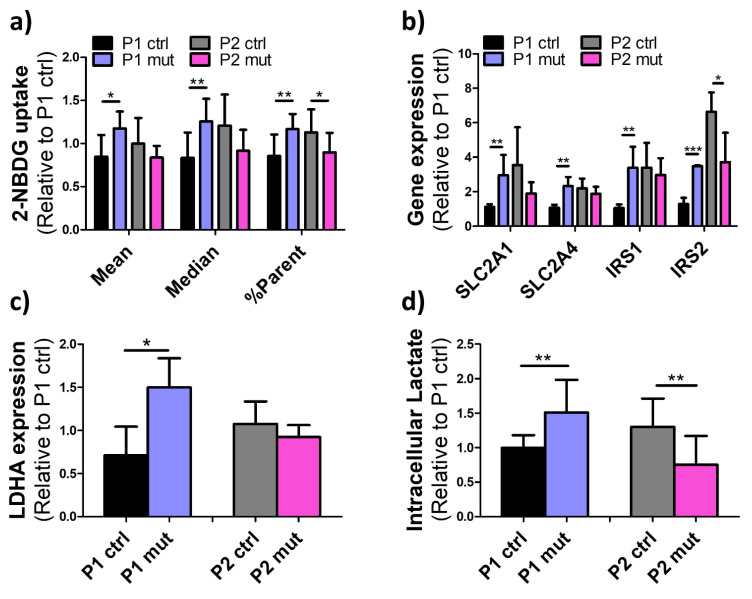
Compensatory glucose metabolism is upregulated in the non-cardiomyopathy patient’s cells. (**a**) Flow cytometry analysis of fluorescent glucose analog (2-NBDG) uptake in iPSC-CMs. Mean and median intensity of 2-NBDG fluorescence intensity and the proportion of 2-NBDG positive cells (%Parent) are shown. Results are relative to one P1 ctrl line. Whereas in P1 a high mutation amount increases cellular glucose uptake, in P2 it decreases it. 3 independent experiments, P1 ctrl *n* = 8, P1 mut *n* = 6, P2 ctrl *n* = 8, P2 mut *n* = 8. (**b**) Expression of genes regulating glucose uptake. Results are relative to P1 ctrl. Three independent experiments, *n* = 6. (**c**) *LDHA* gene expression. Results are relative to one P1 ctrl line. Three independent experiments, *n* = 6. (**d**) Intracellular lactate levels measured with Lactate assay kit. Results are relative to one P1 ctrl line. 2 independent experiments, P1 ctrl *n* = 9, P1 mut *n* = 14, P2 ctrl *n* = 10, P2 mut *n* = 10. Fitting with the glucose usage, a high mutation amount increases lactate levels in P1 cells but decreases them in P2 cells. * *p* < 0.05 ** *p* < 0.01 *** *p* < 0.00. All results are shown as mean +/− SD.

To assess putative changes in glucose metabolism, we analyzed the effect of the m.3243A>G mutations on the iPSC-CMs glucose uptake by flow cytometry of fluorescent glucose analog. Interestingly, while the cells from the CMP patient reduced their glucose uptake upon a high mutation load, further supporting the suppressed metabolism in these cells, the MIDD patient’s (P1) cells with high mutation load increased their glucose uptake (Figure 4a). In line with this, genes involved in regulation of glucose uptake, *SLC2A1* (Solute Carrier Family 2 Member 1), *SLC2A4* (Solute Carrier Family 2 Member 4), *IRS1*(Insulin Receptor Substrate 1), and *IRS2* (Insulin Receptor Substrate 2), were also upregulated in mutant P1 cells but not in mutant P2 cells (Figure 4b). 

When oxidative respiration is not possible, mitochondrial pyruvate is converted to lactate via lactate dehydrogenase A (LDHA), and indeed, increased lactate levels and lactic acidosis are commonly seen in m.3243A>G patients [22]. We thus analyzed the cellular lactate levels and expression of the LDHA gene in patient iPSC-CMs. Consistent with the glucose uptake results, a high amount of the m.3243A>G mutation increased both *LDHA* expression (Figure 4c) and intracellular lactate levels (Figure 4d) in P1 but not in P2 cells with a high mutation load. On the contrary and fitting with the suppressed metabolism and reduced glucose uptake, the intracellular lactate levels decreased in P2 cells with a high m.3243A>G mutation load when compared to control cells from the same patient. 

These results show increased glucose metabolism in the mutant cardiomyocytes of the MIDD patient, whereas similar compensatory increase is not present in the cells of the CMP patient.

### 3.5. High m.3243A>G Mutation Load Elevates ROS Production in the CMP Patient’s Cells

**Figure 5:**
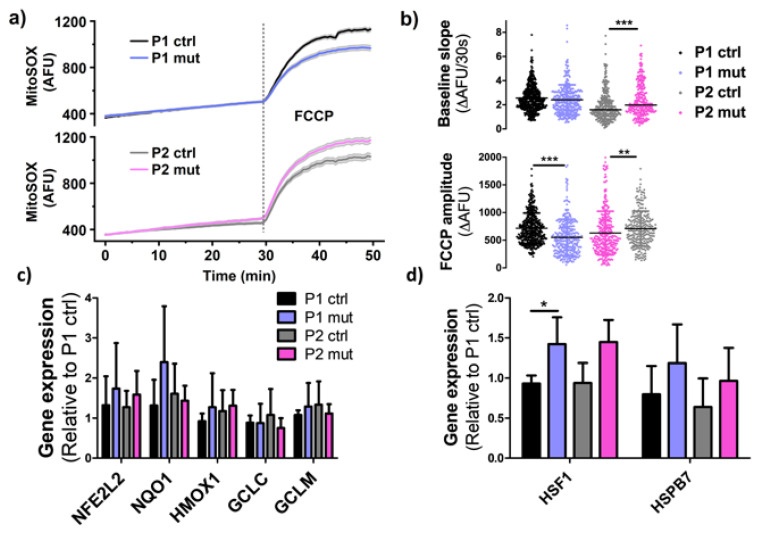
Oxidative stress is increased in the cardiomyopathy patient’s cells. (**a**) Traces of cellular ROS production from developing MitoSOX staining, FCCP introduced at 30 min timepoint. (**b**) Analysis of cellular ROS production by following the intensity of MitoSOX staining at basal state and after FCCP induced maximal respiration P1 ctrl *n* = 513, P1 mut *n* = 325, P2 ctrl *n* = 214, P2 mut *n* = 265 analyzed cells. Mitochondrial ROS production is increased in P2 cells with high mutation load (**c**) Expression of the *NFE2L2* gene (NRF2) and its target genes *NQO1*, *HMOX1*, *GCLC* and *GCLM* relative to one P1 ctrl line. Three independent experiments (*n* = 6). (**d**) Expression of heat shock response genes *HSF1* and *HSPB7*. Relative to one P1 ctrl line. Three independent experiments (*n* = 6). * *p* < 0.05 ** *p* < 0.01 *** *p* < 0.001. All results are shown as mean +/− SD.

Mitochondrial respiration produces reactive oxygen species (ROS) as a by-product of its normal function; further, RC defects can lead to increased ROS production through excess electron leak or electron backflow even with reduced respiration levels [23]. We thus measured the ROS levels in iPSC-CMs both at basal level and after uncoupling with FCCP. In P1, the m.3243A>G mutation level did not affect basal ROS production, and after stress induction by FCCP, ROS levels were decreased in iPSC-CMs with a high mutation load (Figure 5a,b). This is in alignment with the reduced maximal respiration seen in Seahorse analysis (Figure 3a,b). However, in the high mutation amount cells of the CMP patient (P2), ROS production was increased both at the basal level and after FCCP induced uncoupling (Figure 5a,b), despite the reduced respiration rates at both stages (Figure 3a,b).

Cells can defend themselves from excess ROS and one typical process for this is the activation of the NRF2 (Nuclear factor erythroid 2-related factor 2) pathway [24]. We further analyzed expression of both NRF2 itself (*NFE2L2* = Nuclear Factor, Erythroid 2 Like 2) and its target genes: *HMOX1* (Heme Oxygenase 1), *NQO1* (NAD(P)H Quinone Dehydrogenase 1), *GCLC* (Glutamate-Cysteine Ligase Catalytic Subunit), and *GCLM* (Glutamate-Cysteine Ligase Modifier Subunit). However, no significant changes upon increasing mutation levels were seen in cells of either one of the patients (Figure 5c). We further analyzed putative cellular stress response through the heat shock pathway [25]. HSPB7 (Heat Shock Protein Family B member 7), a heat shock protein highly expressed in cardiac tissue, showed a trend for increased gene expression in mutant cells of both patients (Figure 5d), as did the *HSF1* gene (Heat Shock Factor 1), a regulator of the unfolded protein response, and this was significant in the MIDD patient (Figure 5d). 

Taken together, these results reveal increased oxidative stress only in the CMP patient’s cells with high mutation load, yet no significant changes are seen in the oxidative stress defense pathways. Common cellular stress pathways, on the other hand, show an active trend in both patients’ cells with high amount of mutations.

### 3.6. High m.3243A>G Mutation Load Induces Altererations in Calcium-Signaling in the CMP Patient’s Cells

**Figure 6:**
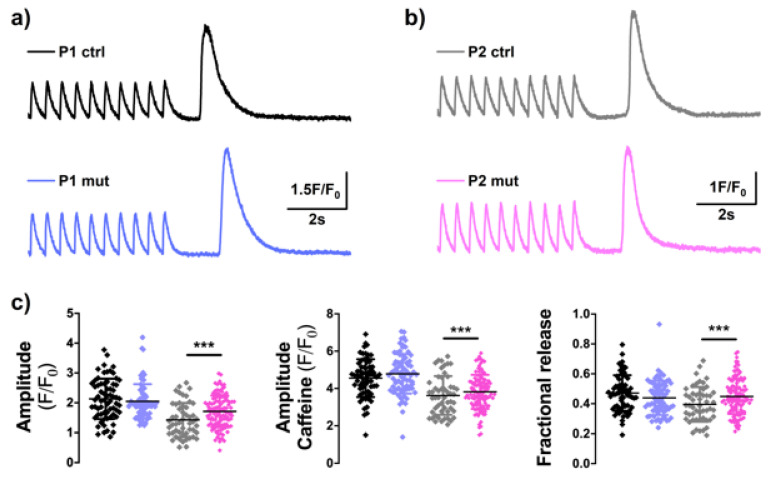
Mutation level affects calcium signaling in the cardiomyopathy patient’s cells. Traces of calcium imaging in iPSC-CMs in basal state and after caffeine stimulation in P1 (**a**) and P2 (**b**). (**c**) Analysis of calcium imaging amplitude in basal state (Ampl.), after caffeine stimulation (Ampl. Caff) and the fractional release calculated from these. High mutation amount increases Calcium transients in P2 cells. P1 ctrl *n* = 65, P1 mut *n* = 75, P2 ctrl *n* = 51, P2 mut *n* = 73 analyzed cells. *** *p* < 0.001. Results are shown as mean +/− SD.

Highly regulated calcium transients (CaT) are an imperative function of cardiomyocytes and enable regulation of their contractile function [26]. Indeed, alterations in calcium signaling reflect arrhythmias and cardiac problems. We did not detect any significant changes in expression of the *ATP2A2* or *RyR2* genes (Figure 1c), both crucial factors of cardiac calcium signaling, in either of the patients. In line with this, we did not find mutation-induced changes in calcium handling in the MIDD patient’s cells (Figure 6). On the contrary, the CMP patient’s (P2) cells with a high m.3243A>G mutation load showed significantly higher calcium transient amplitude when compared to control cells from the same patient, suggesting that in this patient the mutations induced changes to cellular calcium handling (Figure 6). The SR (sarcoplasmic reticulum) -Calcium loading, assessed by caffeine application, was not different between mutant and control cells. Fractional calcium release, evaluated as the ratio of CaT and caffeine pulse amplitudes, was higher in the mutant CMP cells indicating augmented Ca^2+^-release and suggesting alterations in excitation-contraction coupling (Figure 6). 

These data indicate that high mutation amount induces functional consequences for cardiomyocyte contractile function in the mutant cells of the cardiomyopathy patient.

## 4. Discussion

We report here that iPSC-derived cardiomyocytes with mitochondrial mutations retain characteristics of tissue-specific manifestations of the patients’ organs. The genetic background of primary mitochondrial disease has been rapidly unraveling in recent years, however, the pathological mechanisms underlying these common diseases are still mostly unknown. Consequently, the treatment strategies are also sparse and mostly rely on alleviating symptoms with no curative options. Stem cell derived models have revolutionized the use of patient cells in research, helped in functional analysis of cardiomyopathies [27], and have also been widely used in mtDNA disease studies [28]. Yet, while most papers describing mtDNA mutant iPSCs and various differentiated cell types derived from these, report clear respiratory chain deficiency upon high mutation load, the secondary consequences arising from the RC defects described in these papers are varied [28]. This could be somewhat attributed to variation in identity of the studied cell types between different studies, but it may also be in reference to the remarkable clinical variation seen between patients. 

In the current study we utilized m.3243A>G mutation harboring iPSCs to generate mtDNA mutant cardiomyocytes from two patients with different cardiac disease status and examined the effect of high mutation load on the cardiomyocytes of these patients. By comparing the mutant cells to isogenic controls derived from the same patient, we have minimized biological variation and focused only on the mtDNA mutation dependent changes. Independent of the mutation load, all the lines included in this study differentiated to contractile cardiomyocytes that expressed cardiac structural and channel proteins and showed comparable characteristics. Contradictory to some previous studies [29,30], we did not detect any obvious mutation induced defects in cardiac differentiation potential. This may be attributed to different mutation amounts in the studied cells, as in the Yokota et al. study only cells with more than 90% of mutant mtDNA showed these defects [29], and in the Ma et al. study, cell death during differentiation was seen in lines homoplasmic for mutant mtDNA [30], whereas in our study the highest mutation amount was 85%. Our result is consistent with the fact that the m.3243A>G patients do not generally manifest developmental defects [31]. 

We did not detect any mutation-induced changes in the mitochondrial mass or morphology in the iPSC-CMs, suggesting no major effects of the mutations on mitochondrial dynamics or biogenesis. Further, mitochondrial membrane potential, a key indicator of mitochondrial activity [21], was unchanged between different lines, indicating that despite the high, up to 85%, mutation load, the electron transport chain was still functional in the mutant lines. However, in line with several previous studies [29,30], and as a natural and expected consequence of mitochondrial dysfunction, mitochondrial respiration and the proportion of oxygen used for ATP generation by ATP synthase (CV) were reduced in high mutation load lines when compared to control lines. Interestingly, in the mutant cells from the CMP patient, glycolytic activity also seemed suppressed, indicating an overall suppressed metabolism in the mutant iPSC-CMs of this patient. In line with this, the cellular ATP levels were reduced in relation to cells without the mtDNA mutations. 

The heart has a high rate of ATP turnover, which is required to maintain its continuous mechanical work. ATP deficiency can therefore directly affect cardiac contractile function and the reduced ATP levels in the CMP patient’s cells are likely to induce functional consequences. In line with this, we detected slightly increased cytoplasmic Ca^2+^ transient amplitudes in the mutant cells of the CMP patient indicating functional consequences for the contractile properties of these cells. Cardiac contractility is regulated by changes in intracellular Ca^2+^ concentration and regular heartbeat requires tightly controlled calcium homeostasis and sufficient transients in Ca^2+^ levels between systole and diastole [32]. Mitochondria can regulate cellular calcium homeostasis in multiple ways. The reduced ATP levels in this patient’s cells may either directly underlie the changes in contractile properties, or alternatively, for example altered mitochondrial calcium intake through the mitochondrial Ca uniporter (MCU) could result in extensive mitochondrial calcium intake affecting cytoplasmic calcium transients [33]. Interestingly, the ATP levels were unaltered in the MIDD patient‘s cells with a high mtDNA mutation load and consequently no changes were seen in the cellular calcium homeostasis, suggesting that reduced ATP levels underlie the contractile changes in the CMP patient’s cells. 

Signs of oxidative stress were seen in the CMP patient’s cells with high mtDNA mutation amounts. Elevated ROS levels are common in mitochondrial dysfunction despite reduced respiration, as dysfunctional electron transport chain can increase ROS production either through reverse electron transport, altered proton motive force or changes in the ratio of reduced and oxidized nicotinamide adenine dinucleotide (NAD^+^/NADH) [34]. Complex I is one of the sites for mitochondrial ROS production. When membrane potential is normal, but RC function is compromised, the relative NAD^+^ level decreases and the coenzyme flavin mononucleotide of CI becomes over-reduced and cannot accept further electrons. This leads to premature escape of electrons and production of superoxide [35]. Interestingly, an increase in cellular ROS was not detected in the mutant MIDD patient’s cells, which manifested CI deficiency. Complex I deficiency is common in patient tissues and has also been seen in several previous m.3243A>G iPSC studies [16,29,36]. We suggested earlier that this CI deficiency could be an initial protective mechanism upon RC dysfunction aimed at reducing ROS produced by CI [16]. With CI downregulated, the RC can still function through increasing CII activity, a compensatory mechanism commonly seen upon mitochondrial dysfunction. This initial defensive downregulation of CI could explain why the ROS levels were not increased in the mtDNA mutant MIDD cells. Why this protective mechanism was not activated in the CMP patient’s cells remains unknown. However, as a result, oxidative stress is increased in the CMP patient’s cells with high amount of mutations, which may further contribute to the functional decline of these cells.

**Figure 7:**
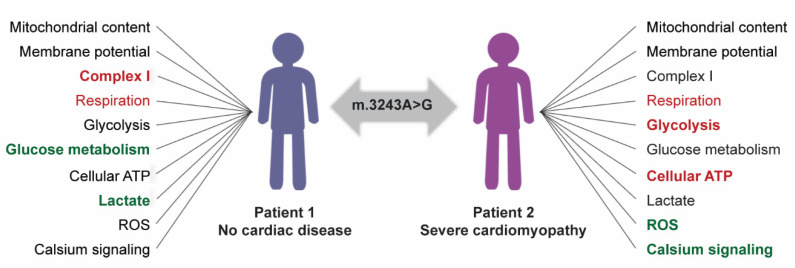
Summary of the effects of high m.3243A>G mutation load on two independent patients. Decreased functions indicated in red, unchanged in black, and increased in green fonts. The different responses between patients indicated in bold. mt = mitochondrial.

The two patients included in our study, show different responses to a high m.3243A>G mutation load and RC dysfunction in their cardiac cells (Figure 7). While respiratory metabolism is down in both patients, the patient with MIDD responds with downregulating complex I and increasing glycolytic metabolism. These initial responses result in maintained ATP levels despite a high mutation load. Further, the cytoplasmic calcium transients are not altered in this patient’s cells. On the contrary, similar compensatory mechanisms do not take place in the CMP patient’s cells and as result, cellular ATP levels are decreased in cells with a high amount of mutations. Further, the cellular calcium transients are affected in cells with a high amount of mutant mtDNA, suggesting altered contractile function in the CMP patient’s cells. 

Our findings support the hypotheses that a high mtDNA mutation load alone is not sufficient to trigger clinical heart disease in mtDNA disease patients; but, other adaptative metabolic pathways, either expose or protect the patient from cardiac manifestations. Currently, due to the severity of the heart symptoms, regular cardiac follow-ups are recommended for all m.3243A>G patients [37]. Understanding these additional factors would aid in early identification of those patients that will develop heart defects. Moreover, understanding the initial mechanisms leading to these varying responses between patients could allow for modification of the responses and even affect the outcome of the cardiac phenotype. Further studies are needed in additional patient material to identify these mechanisms and to understand whether they are uniform between different patients or whether additional heterogeneity is seen in additional study subjects.

The differentiation protocol used in this study results in fairly immature cells that resemble more fetal than adult cells and in line with this fetal nature, the metabolism of these cells is still quite glycolytic and does not completely rely on mitochondrial fatty acid oxidation [38,39]. Thus, our results are not fully comparable to the situation in adult patients’ hearts, but rather resemble early responses to the RC defect in maturing cardiac cells. Whether similar compensatory mechanisms that are seen here can sustain functionality in more mature cardiomyocytes remains to be seen. Studies in more mature cells are needed to fully understand the situation in adult patients’ hearts. New methodology in cardiac cell differentiation, e.g., co-cultures of various cardiac cell types and different microtissue cultures, which have been shown to improve functionality and mitochondrial metabolism of iPSC-derived cardiomyocytes, will hopefully aid in this in the future [40,41]. Recent advances in gene-editing technologies have opened new opportunities in targeted modification of mitochondrial genome [42,43], and animal models with mtDNA mutations can soon aid in studies on mitochondrial DNA defects. However, these cannot recapitulate the patient-to-patient variation and thus do not enable questions on clinical variability. Thus, studies on patient derived material remain critical.

## 5. Conclusions

In conclusion, our results show that cells from different patients respond differently to a high m.3243A>G mutation load. Reduced ATP levels in the CMP patient’s cells lead to altered calcium handling, while compensatory metabolic activity is able to retain normal functionality in MIDD patient’s cells with equally high mutation loads. Thus, a high mtDNA mutation amount alone is not sufficient to induce functional changes in cardiac cells.

## Data Availability

All data supporting the conclusions are available upon request from the corresponding author.

## Supplementary Materials

The following supporting information can be downloaded at: www.mdpi.com/article/10.3390/cells11162593/s1, Figure S1: Uncropped western blots.

## Appendix A. Additional Methodological Details

**Table A1:** List of antibodies used in the study.

Type	Antibody	Origin	Company (Product Code)	Application (Dilution)
Primary	Cardiac Troponin T	Goat	Abcam (ab64623)	IF (1:500)
	TOM20	Rabbit	Cell Signaling (42406)	IF (1:200) WB (1:500)
	Porin	Rabbit	Abcam (ab15895)	WB (1:1000)
	Total OXPHOS Human WB antibody	Mouse	Abcam (ab110411)	WB (1:1000)
	Myosin Heavy Chain	Mouse	Millipore (05-716)	IF (1:400)
	α-Tubulin	Mouse	Abcam (ab40742)	WB (1:2000)
Secondary	Anti-mouse, Alexa fluor 488	Donkey	Invitrogen (A21202)	IF (1:300)
	Anti-goat, Alexa fluor 568	Donkey	Invitrogen (A11057)	IF (1:300)
	Anti-goat, Alexa fluor 488	Donkey	Invitrogen (A11055)	IF (1:300)
	Anti-rabbit, Alexa fluor 594	Donkey	Invitrogen (A21207)	IF (1:300)
	Anti-mouse, biotinylated	Horse	Vector Laboratories (BA-2000)	WB (1:3000)
	Anti-rabbit, biotinylated	Goat	Vector Laboratories (BA-1000)	WB (1:5000)

**Table A2:** Primer sequences used in qPCR.

Gene	Sequence 5′->3′	Reference
APP forward	GCCTGCCTGATCCTCCAAAT	
APP reverse	AGGGTAGCGGATGATTCAGCC	
CYTB forward	TGTGTGCTCTCCCAGGTCTA	
CYTB reverse	CAGTTCTGGATGGTCACTGG	
ANP forward	GATCTGCCCTCCTAAAAAGCAA	
ANP reverse	ATCTCCGCAGGCTCCGA	
ATP2A2 forward	GCGGGCAATCTACAACAACA	
ATP2A2 reverse	GAGCAGCTGAACAGGAATCAAA	
RYR2 forward	AACTACTGTCCAAGCAGAGGAAAAG	
RYR2 reverse	CCGATGCCTTGGCAGATTAT	
SLC2A1 forward	GTCTGGCATCAACGCTGTCTT	
SLC2A1 reverse	CTGCTCGCTCCACCACAAAC	
SLC2A4 forward	CGACCAGCATCTTCGAGACAG	
SLC2A4 reverse	TCCACCAACAACACCGAGACC	
IRS1 forward	AACTGGACATCACAGCAGAATGAA	
IRS1 reverse	CAAACTGAAATGGATGCATCGTACC	
IRS2 forward	GCCACCATCGTGAAAGAGTGA	
IRS2 reverse	TTGCCTTGTTGGTGCCTCAT	
LDHA forward	AGCCCGATTCCGTTACCT	[44]
LDHA reverse	CACCAGCAACATTCATTCCA	[44]
GAPDH forward	AATGAAGGGGTCATTGATGG	
GAPDH reverse	AAGGTGAAGGTCGGAGTCAA	
TUBA3 forward	CATTGCCAATCTGGACACC	
TUBA3 reverse	GCAACAACCTCTCCTCTTCG	
HSF1 forward	TGAGAATGAGGCTCTGTGG	
HSF1 reverse	CTGAATGAGCTTGTTGACGA	
HSPB7 forward	AGCGAAGGTGTTCATGACG	
HSPB7 reverse	CCTATGAGTTTGCGGTGGAC	
NFE2L2 forward	GGTTGCCCACATTCCCAAATC	[45]
NFE2L2 reverse	CAAGTGACTGAAACGTAGCCG	[45]
HMOX1 forward	TTCAAGCAGCTCTACCGCTC	[45]
HMOX1 reverse	GAACGCAGTCTTGGCCTCTT	[45]
NQO1 forward	TGCAGCGGCTTTGAAGAAGAAAG	[46]
NQO1 reverse	TCGGCAGGATACTGAAAGTTCGC	[46]
GCLC forward	ATGGAGGTGCAATTAACAGAC	[46]
GCLC reverse	ACTGCATTGCCACCTTTGCA	[46]
GCLM forward	GCTGTATCAGTGGGCACAG	[46]
GCLM reverse	CGCTTGAATGTCAGGAATGC	[46]

**Table A3:** Taqman primers and probes used for the determination of the m.3243A>G mutation frequency.

Forward primer	5′-CCACCCAAGAACAGGGTTTG-3’
Reverse primer	5′-GGAATTGAACCTCTGACTGTAAAGTTT-3’
3243A probe	VIC-AGATGGCAGAGCCCGGTA-MGB
3243G probe	FAM-AGATGGCAGGGCCCGGTA-MGB
