# Supplementary material for: Varied Responses to a High m.3243A>G Mutation Load and Respiratory Chain Dysfunction in Patient-Derived Cardiomyocytes
Source: Cells. 2022 Aug 19;11(16):2593. doi: 10.3390/cells11162593 (PMC9406376; doi:10.3390/cells11162593)
Supplement: Supplementary file 1 [file cells-11-02593-s001.zip › cells-1805923-supplementary.pdf]

Supplementary material

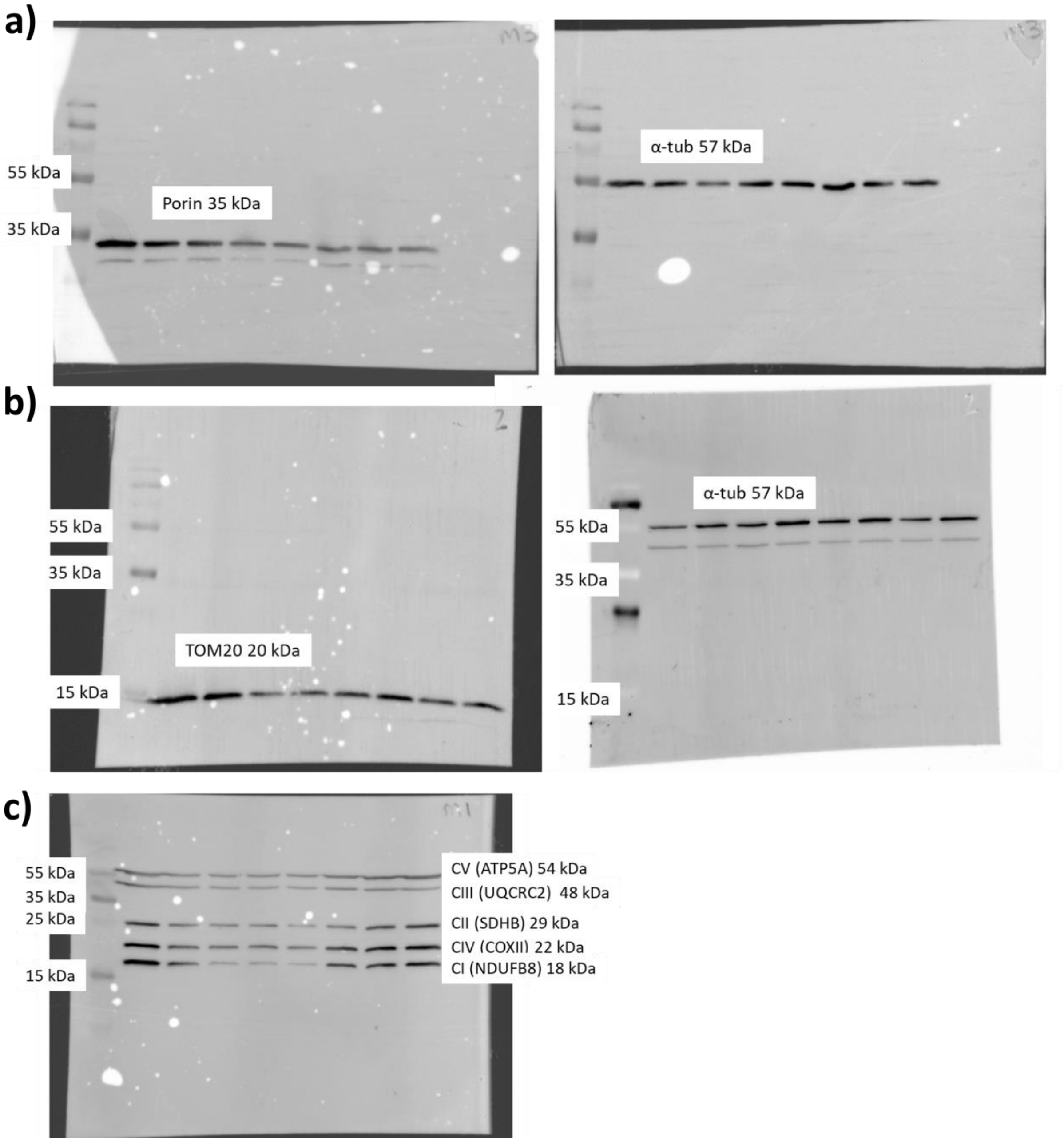

Figure S1. Uncropped Western blots (representative images). a) Porin (left) and α-tubulin (right) in the same blot . b) TOM20 (left) and α-tubulin (right) in the same blot. c) Total OxPhos Human wb antibody (CI-CV). Molecular weight marker: PageRuler Plus Prestained Protein ladder (Thermo Scientific, #26619).
